# Associations between nesting, stereotypy, and working memory in deer mice: response to levetiracetam

**DOI:** 10.1007/s43440-023-00484-2

**Published:** 2023-04-13

**Authors:** Bianca Hurter, Shannon L. Gourley, De Wet Wolmarans

**Affiliations:** 1 Department of Pharmacology, Faculty of Health Sciences, Centre of Excellence for Pharmaceutical Sciences, North-West University, Potchefstroom, 2520 South Africa; 2 Departments of Pediatrics and Psychiatry, Emory School of Medicine, Atlanta, USA; 3 Children’s Healthcare of Atlanta, Atlanta, USA; 4 Emory National Primate Research Center, Emory University, Atlanta, USA

**Keywords:** Deer mouse, Stereotypy, Nest building, Barnes maze, T-maze, Alternation, Working memory

## Abstract

**Background:**

Some deer mice *(Peromyscus maniculatus bairdii)* exhibit various phenotypes of persistent behaviors. It remains unknown if and how said phenotypes associate with early-life and adult cognitive perturbations, and whether potentially cognitive enhancing drugs might modify such associations. Here, we explored the longitudinal relationship between early-life behavioral flexibility and the expression of persistent behavior in adulthood. We also investigated how said phenotypes might associate with working memory in adulthood, and how this association might respond to chronic exposure to the putative cognitive enhancer, levetiracetam (LEV).

**Methods:**

76 juvenile deer mice were assessed for habit-proneness in the Barnes maze (BM) and divided into two exposure groups (*n* = 37–39 per group), i.e., control and LEV (75 mg/kg/day). After 56 days of uninterrupted exposure, mice were screened for nesting and stereotypical behavior, and then assessed for working memory in the T-maze.

**Results:**

Juvenile deer mice overwhelmingly utilize habit-like response strategies, regardless of LNB and HS behavior in adulthood. Further, LNB and HS are unrelated in terms of their expression, while LEV reduces the expression of LNB, but bolsters CR (but not VA). Last, an increased level of control over high stereotypical expression may facilitate improved working memory performance.

**Conclusion:**

LNB, VA and CR, are divergent in terms of their neurocognitive underpinnings. Chronic LEV administration throughout the entire rearing period may be of benefit to some phenotypes, e.g., LNB, but not others (CR). We also show that an increased level of control over the expression of stereotypy may facilitate improved working memory performance.

**Supplementary Information:**

The online version contains supplementary material available at 10.1007/s43440-023-00484-2.

## Introduction

Persistent and repetitive behaviors are common in neuropsychiatric conditions, e.g., obsessive–compulsive disorder (OCD) and its other related disorders, autism spectrum disorder, behavioral addictions like gambling disorder, and attention-deficit/hyperactivity disorder [1]. Significant advances have been made to understand the neurocognitive underpinnings of these behaviors. Current research implicates cortico-striatal-thalamo-cortical involvement [2, 3], with cognitive theory variably pointing to dysfunctional processes that regulate, among others, habit formation [4, 5], impulse control [6–8], reward feedback processing [9–11], and cognitive flexibility [12–14]. The contribution of these and other constructs to the development of persistent and repetitive behaviors are likely unique to different conditions, which clouds our understanding of how these behaviors develop over time.

OCD is diagnosed in up to 3% of children and adolescents [15]. Diagnosis can be especially difficult, since many of the symptoms that are assessed during evaluation correspond with behavioral traits that often present in children, e.g., highly ritualized and rigid habit-like behaviors [16, 17]. Further, it is uncertain if and how such traits may predict and ultimately contribute to a clinical diagnosis of OCD in some but not others [18–20]. In terms of clinical progression, neurocognitive processes that regulate the relationship between goal-directed and habitual responses have been proposed to play a role in obsessive–compulsive symptom manifestation [21]. For example, individuals that show a bias toward habitual behavioral engagement [22] or that present with deficits in goal-directed action-outcome valuation [23, 24], both of which may also be markers of impaired cognitive flexibility [25], are at increased risk to develop OCD.

High-dose selective serotonin reuptake inhibitor (SSRI) treatment is the mainstay of current treatment approaches [26]. Meanwhile, cognitive strategies mainly involve behavioral therapy and exposure–response prevention [27]. In both instances, treatment responses are less than adequate, highlighting a need for alternative options [28]. To this end, early-life pharmacotherapeutic strategies that may target distinct psychobiological processes that can, in some cases, contribute to the development and prognosis of compulsivity, might be fruitful. From this theoretical perspective, levetiracetam (LEV), clinically used for the treatment of partial and generalized epilepsy [29], shows promise. Although most data that point to the neurocognitive effects of LEV, e.g., improvement in working memory, verbal skill, and visual feedback processing, have been generated in epilepsy cohorts [29–31], some results from studies in other clinical cohorts, e.g., patients suffering from Alzheimer’s disease [32], and non-clinical samples have also been informative. For example, a single dose of LEV enhances executive functioning on the levels of working memory, task planning and decision making [33].

Subpopulations of deer mice *(Peromyscus maniculatus bairdii)* that are bred, reared, and housed under standard laboratory conditions variably and spontaneously present with distinct, but equally persistent and repetitive behavioral phenotypes, large nest building behavior (LNB) and high motor stereotypy (HS) [34]. HS behavior in turn can be classified into either vertical jumping activity (VA) or patterned cage running activity (CR). These behaviors are entirely non-induced, occur in animals of both sexes, and respond to chronic high-dose oral exposure to the SSRI, escitalopram [35], making these mice an interesting model organism for studying compulsive-like behaviors. In this work, we explored the longitudinal relationship between a potential early-life marker of habit-proneness in the Barnes maze (BM) and LNB and HS (on the levels of VA and CR) in adulthood. We further sought to understand if and how these behaviors associate with each other and with working memory, as assessed in the T-maze. Last, we investigated if LNB, VA, and CR, as well as potential relationships with working memory, would respond to chronic LEV exposure, administered throughout the rearing period.

## Materials and methods

### Study layout

**Fig. 1:**
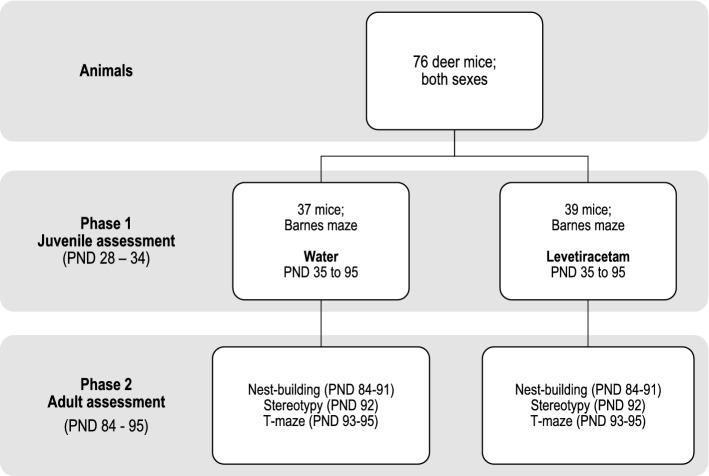
Schematic representation of study layout; *PND* postnatal day. All mice received either normal water or levetiracetam (LEV) for an uninterrupted period of 60 days until study endpoint and throughout all adulthood behavioral experiments. Said exposure was initiated after completion of the baseline Barnes maze assessment

The present work was divided into two phases, Phase 1 (juvenile) and Phase 2 (adult) (Fig. 1). First, 76 mice were assessed on the Barnes maze (BM) from postnatal day (PND) 28–35. Next, mice were divided into two groups, control (CTRL) and LEV (CTRL: *n* = 37, 20 females, 17 males; LEV: *n* = 39, 20 females, 19 males) and treated until PND 95. From PND 84–95, all mice underwent sequential behavioral testing, nest building (PND 84–91), stereotypy assessment (PND 92), and T-maze assessment (PND 93–95).

### Mice

*Peromyscus maniculatus bairdii* were acquired from the DSI/NWU Vivarium at North-West University, South Africa (South African Veterinary Council registration number: FR15/13158; AAALAC accreditation file #1717). All procedures were approved by the AnimCare Research Ethics Committee of the NWU (approval number: NWU-00423–21-A5) and complied with the South African National Standard for the Care and Use of Animals for Scientific Purposes (SANS 10,386:2021). The number of animals included in this investigation was broadly based on extensive prior investigation where we showed that at least 30–45% of mice develop LNB [34, 36–39] and motor stereotypies [40–43] in adulthood.

Experimental subjects were chosen by means of a randomized number system from the litters of at least 20 unique breeding pairs. After selection, animals were ear-tagged for identification purposes and, except if stated otherwise, housed in pairs of same-sex animals throughout the remainder of the investigation. Cages [35 cm (l) × 20 cm (w) × 13 cm (h); Techniplast® S.P.A., Varese, Italy] were automatically climate-controlled and kept ambient at 23 °C, a relative humidity of 60%, and on a 12-h light/dark cycle (06h00/18h00). Food and water (or drug solution) were provided *ad lib* throughout all phases of the study, while cages were cleaned and new corncob bedding supplied, weekly. Except if stated otherwise, all cages were supplied with paper towel as a form nesting material and equipped with a piece of white polyvinyl chloride (PVC) pipe [10 cm (l) × 5 cm (Ø)] as a form of environmental enrichment.

### Drug exposure

On the day following the last BM probe trial (PND 35), CTRL (normal tap water) or LEV (BLD Pharma®, Shanghai, China) exposure was initiated via the drinking water and continued through the end of the study period. Levetiracetam was prepared at a concentration of 30 mg/100 mL to deliver an approximate 24-h dose of 75 mg/kg/day; Sanchez, Zhu [32], de Ridder, Mograbi [40]. Since the drug is freely soluble in water, no other additives were introduced to the solution. Drug solutions were provided in the standard, amber-colored polycarbonate Techniplast® system bottles. Drug exposure via the drinking water is preferred in this model system, as opposed to intraperitoneal injection or oral gavage, due to the long period of drug exposure. Fresh drug solutions were constituted every day to ensure optimal drug delivery. Still, LEV is stable for up to 14 days in aqueous solution [44]. Chosen concentrations were based on the average daily fluid intake of adult deer mice (0.25 mL/g/day; de Brouwer, Fick [34]). To confirm, the fluid consumption of LEV-exposed mice was recorded daily and compared to that of deer mice in the laboratory that only received water (Supplementary data). Importantly, since animals were housed in pairs to avoid the potential influence of social isolation-related anxiety on the behavioral output [45], the exact amount of water or LEV solution ingested by each mouse in a cage, could not be determined accurately. Rather, since mice dehydrate rapidly in the absence of adequate fluid intake [46], measurements of cage consumption were applied together with daily health checks to verify optimal drug intake.

### Behavioral assessment—general background

Since 76 mice were tested in behavioral experiments, procedures were timed to enable a sequential experimental approach. Specifically, animals that were born at different time points entered into the behavioral assessment procedures according to their respective ages, as indicated for each assessment in in Fig. 1**.** This accommodated for the maximum availability of research infrastructure and ensured that each animal went through the same chronological experience, according to age. Importantly, for the purposes of this paper, the methods have been discussed in brief, only. Please refer to the supplementary material for detailed descriptions.

### Barnes maze assessment

BM experiments were conducted in the dark cycle, between 19:00 and 01:00. A maximum of 15 mice were assessed on any given night, while both CTRL- and LEV-exposed mice were assessed during the same hours of the dark cycle. A small, walled-off Barnes maze, adapted from O'Leary and Brown [47], was used. The maze was constructed from white Plexiglas® and consisted of an octagon-shaped floor with a diameter of 70 cm, walled off on each side (50 cm high). 16 circular holes (4 cm in diameter) were equally spaced around the perimeter of the maze floor. The maze was raised to a height of 30 cm above floor level to allow for the placement of a movable escape box (15 cm (l) × 15 cm (w) × 20 cm (h)). When the escape box was attached to the BM, all other holes were closed off. A separate floorless starting compartment (10 cm × 10 cm × 15 cm) was placed in the center of the BM at the onset of testing. Except if stated otherwise, all phases of the Barnes maze experiment were conducted under bright white light (10 000 lx), while white noise was played in the background at a sound level of 80 dB to bolster escape-related behavior [48].

The BM assessment was conducted as previously described [47], with minor modification to allow for the assessment of juvenile deer mice. Briefly, mice were habituated, trained, and tested over three separate stages: (A) habituation stage, (B) acquisition stage, and (C) probe stage. All trials were video recorded to allow for post-test scoring and automated tracking of ambulatory behavior (Ethovision® XT 16; Noldus Information Technology®, Wageningen, The Netherlands).

For the habituation stage (A) mice were first introduced under a glass beaker over the area of the escape hole in the absence of any aversive stimuli. Then, 25 min later, mice were allowed to freely explore the entire maze. The acquisition stage (B) comprised four training days with two trials per day. In this phase, mice had to learn the location of the escape hole while being exposed to an aversive environment. Mice were accepted to have learned the location of the escape hole if they made four or more successful attempts at reaching the hole or entered the burrow over the eight stage (B) trials, collectively. In the probe stage (C), animals were assessed for habit-like behavior, i.e., returning to the proximity area around, or attempting to enter the hole to which the escape box was previously attached, but was now closed off. The probe trial phase was conducted over 2 days of testing. More entries toward the target area in the first trial compared to the last, was regarded as behavioral engagement regulated by goal-directed processing, while a similar number of approaches toward the target area through the final stage (C) trial, was regarded as habit-like.

### Nesting assessment

**Fig. 2:**
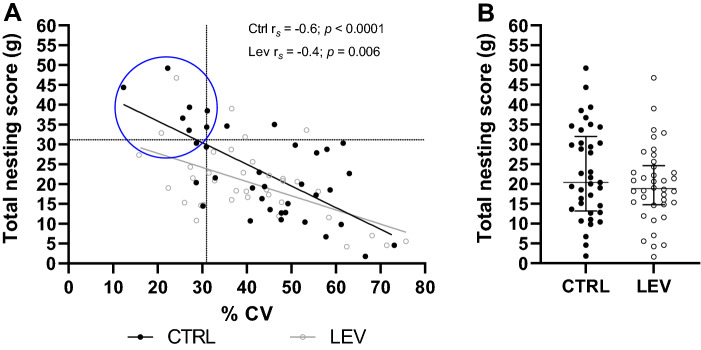
**A** LNB cohort selection (in the blue oval), for the control (CTRL)- and levetiracetam (LEV)-exposed mice based on the total nesting scores in gram and the % coefficient of variance (CV) pertaining to the daily nesting scores (as a measure of behavioral persistence over subsequent testing days) calculated for animals in the CTRL-exposed group; **B** Total nesting scores in gram, for CTRL and LEV-exposed groups; Spearman’s correlation; data are median with 95% CI; Dotted lines in **A** indicate the 75th and 25th quartiles of distribution for the total nesting scores and % CV, respectively; CTRL: *n* = 37, LEV: *n* = 39

All 76 mice were assessed for nest building expression over one week once animals reached the age of 84 days [34]. For this assessment, mice were housed singly. On each day, a weighed excess of sterile cotton wool was introduced into the roof of each cage. On every subsequent day, built nests were removed and the remaining cotton wool weighed. Where needed, additional cotton wool was supplied. Thus, mice had access to nesting material for nearly 24 h of each day. Identification of normal nesting (NNB) and LNB was based on the extreme ends of the distribution of all total nesting scores generated by mice in the CTRL- and LEV-exposed groups [34]. Further, a second criterion, that is persistence, was applied to classify LNB. Thus, only animals that generated total nesting scores that clustered within the upper 75^th^ percentile of the distribution, and the lowest quartile of distribution with respect to the variance in the daily nesting scores (as reflected by the percentage coefficient of variance; % CV), are classified as LNB-expressing animals (Fig. 2A).

### Stereotypy assessment

On the first day following the conclusion of the nest building assessment, all mice were assessed for stereotypical behavior according to a previously published protocol [40], slightly modified for the purposes of the present investigation. Briefly, each mouse underwent a single 12-h stereotypy assessment in its own behavioral testing cage [21 cm (l) × 21 cm (w) × 35 cm (h); Accuscan® Inc., Columbus, Ohio, USA]. Food and water (or drug solutions) were provided *ad lib* throughout the 12-h assessment session.

### T-maze assessment

Assessment of arm alternation behavior in a T-maze is often used to investigate processes related to working memory [49]. Here, we applied a modified version of the test used by Gerlai [50]. Animals were trained to enter one arm of the maze, prior to alternation testing, to test for habit-like behavior. Briefly, the maze was shaped in the form the letter ‘T’ (stem and arm dimensions 30 cm × 6.5 cm; wall height: 30 cm). When needed, each of the respective arms could be closed off by a manually operated door [51]. The ambulatory activity of mice was also analyzed with Ethovision® XT 16 software.

The T-maze assessment was also conducted over three stages: (A) naturalistic arm preference testing, (B) cued forced-arm entry training, and (C) probe test. Stages were separated by 24 h, with stage (A) commencing 24 h after the onset of stereotypy assessment. All T-maze experiments were conducted between 19:00 and 01:00 under dim red light. During stage (A), mice were allowed a single 15-min session to explore the maze, or to make a maximum of ten arm entries. The most preferred arm was determined based on the majority arm choice. For the cued forced-arm entry stage (B), mice were forced to enter the least preferred arm (i.e., ‘forced arm’) as determined in stage (A), as many times as they could within 30 min, each entry separated by a 30-s inter-trial interval. During the probe test stage (C), mice were able to access the entire arena again. The arm that was cued in stage (B) remained thus, with the opposite arm being non-cued. Mice were again allowed to enter the maze as many time they could within 30 min. Higher, as opposed to lower post-training alternation scores are indicative of better working and spatial memory recall.

### Statistical analyses

Statistical analyses were performed, and graphs drawn with GraphPad® Prism® version 9. Associations between the probe trial behavior of juvenile mice (trial vs. target approach) and the other behavioral outcomes of this investigation (nesting expression, stereotypy, and T-maze alternation, etc.), were explored by multiple Spearman’s correlations. The same analysis was used to determine the association between total nesting score and the percentage coefficient of variance with respect to daily nesting scores in adult mice (i.e., an indicator of behavioral persistence over subsequent testing days) exposed to either CTRL or LEV (Fig. 2). Descriptive statistics were applied to determine the upper 75th and the lower 25th quartiles of distribution for each parameter, respectively. This was used to compare the expression of LNB in CTRL and LEV-exposed mice. For comparisons of the total nesting scores, stereotypical intensity and time spent engaging in HS behavior, and T-maze alternation scores generated by CTRL- and LEV-exposed mice, unpaired Mann–Whitney *U-*tests were run. Significance was regarded as *p* < 0.05.

## Results

### Summary of animals that completed the respective assessments

Since this study followed a longitudinal approach, some animals failed to complete the entire battery of assessments. As such, statistical analyses are based on the data of animals that completed the respective assessments (Supplementary Table I), while the correlational data are only reflective of animals that generated data for both relevant parameters. The behavioral measures for the CTRL and LEV groups, respectively are reported in the Supplementary Tables II & III.

### Barnes maze assessment

Of the 76 juvenile mice assessed for BM behavior, 10 mice failed to satisfy the criteria for successful learning (meaning, they failed to locate the escape port more than 50% of the time). Thus, the probe test data were not considered. Of the remaining 66 mice, only 6 mice fulfilled the criterium for goal-directed responding (colored lines; Supplementary Table II), with the remaining mice all generating neutral to positive slope scores (meaning, they persisted in familiar strategies even when they were not productive). Of these, multiple Spearman’s correlations failed to reveal significant relationships with any of the parameters assessed in adulthood, or with the number of successful training trials prior to the test (not shown).

### Nesting behavior of CTRL- and LEV-exposed mice

Total nesting scores correlated negatively with the variance in daily nesting scores in both CTRL- and LEV-exposed mice (Fig. 2A, CTRL: *r*_*S*_ = − 0.60; *p* < 0.0001; LEV: *r*_*S*_ = – 0.44; *p* = 0.006). Based on the statistical cut-off criteria used to identify LNB-expressing mice in the CTRL-exposed group, eight LNB-expressing mice were identified from the pool of 37 (22%), while only two mice in the LEV-exposed group (5%) fulfilled the same criteria.

In terms of the average total nesting scores generated by CTRL- and LEV-exposed mice (Fig. 2B), no significant difference between the median values of the different groups was shown (*p* = 0.32, 95 CI: – 8.1; 2.5, *U* = 608.5, *N*_1_ = 37, *N*_2_ = 38).

### Stereotypical behavior of CTRL- and LEV-exposed mice

**Fig. 3:**
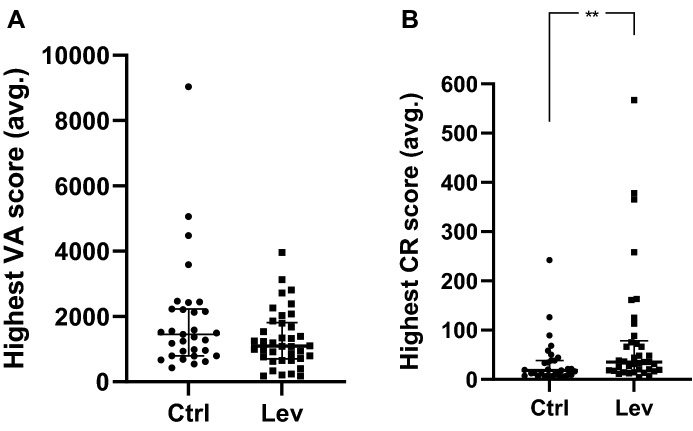
Highest average vertical jumping **(A)** and pattern running **(B)** activity scores for the control (CTRL)- and levetiracetam (LEV)-exposed groups; Mann–Whitney *U-*test; **p < 0.01; CTRL: *n* = 27, LEV: *n* = 25

The median values of the highest average VA scores generated by mice in the CTRL- and LEV-exposed groups were similar (Fig. 3A; *p* = 0.12, 95 Cl: – 734.0; 92.0, *U* = 461.0, *N*_1_ = 31, *N*_2_ = 38). In contrast, LEV exposure significantly increased the expression of CR activity, compared that of CTRL-exposed mice (Fig. 3B**;** *p* = 0.0078, 95 Cl: 4.00; 29.0, *U* = 370.5, *N*_1_ = 31, *N*_2_ = 38).

In terms of the time that NS and HS animals spent engaging in HS expression, LEV-exposure had no effect on the result (*p* = 0.92, *U* = 581.5, *N*_*1*_ = 31, *N*_2_ = 38).

### T-maze alternation behavior of CTRL- and LEV-exposed mice

Alternation scores generated by CTRL- and LEV-exposed mice that completed the assessment, did not differ significantly (*p* = 0.76; *U* = 408.5, *N*_1_ = 33, *N*_2_ = 26).

### Associations between nesting, stereotypy, and T-maze alternation in adult mice

To determine whether any relationships exist between nesting behavior, stereotypical expression, and T-maze alternation in adult mice, the following Spearman’s correlations were run: (i) total nesting scores vs. stereotypical expression (both intensity and time spent executing HS behavior across both VA and CR), (ii) total nesting scores vs. alternation scores, and (iii) stereotypical expression (both intensity and time spent executing HS behavior across both VA and CR) vs. alternation scores. Statistics are provided in Supplementary Table IV.

## Discussion

The main findings of the present work are (1) performance in the BM of juvenile *P. maniculatus bairdii* mice is not related to LNB or stereotypy in adulthood; (2) chronic LEV exposure from a juvenile age until adulthood decreases LNB expression without affecting overall nesting scores, but increases CR activity; (3) stereotypical expression, notably so CR, and nesting expression are divergent persistent behavioral phenotypes; and (4) LEV exposure generally improves the relationship between working memory and stereotypical engagement.

One aim of this work was to explore the potential longitudinal relationship between habit-like behavior early in life and compulsive-like behavior in adulthood. We used a BM task, reasoning that repeated exposure to an aversive environment, paired with a means to escape, may be an appropriate method to facilitate habit learning, as reflected by continued approach to the escape location even when it was no longer available. If we accept this premise, our results indicate that nearly all mice in this large study utilized habit-like behavior. Consistent with this notion, probe test behavior was overwhelmingly uncoupled from initial escape acquisition, suggesting that it was driven by factors other than escape learning, such as behavioral automaticity. In operant conditioning procedures, young rats favor habit-based response strategies even under conditions that induce goal-seeking behavior in adults, suggesting that young organisms are more prone to use habit-based action strategies [52]. Allen, Heaton [53] capitalized on this propensity to identify highly habit-prone mice for later selective breeding for habit bias. Relatedly, Moin Afshar, Keip [54] revealed that the capacity for goal-based action strategies develops across juvenile and adolescent development, crystalizing only in young adulthood [54]. Thus, the response strategies observed here may reflect the strong tendency of young animals to utilize habit-based behaviors. Indeed, juvenile *P. maniculatus bairdii* may be a valuable tool to understand the neurobiology of habit formation and adherence in young developing mammals. This topic is grossly under-studied, as most investigations instead focus on the development of competing goal-directed response systems as organisms mature into adulthood.

A second consideration that deserves attention relates to prior work using the Morris water maze, revealing that mice postpone or refrain from active responding in aversive environments, since they tend to freeze under aversive circumstances [55]. Such a manner of responding could well have influenced training success in the present paradigm. Irrespective, what remains unknown is whether the results reported here are borne from such methodological constraints, or whether it is reflective of potential deficits in executive functioning, possibly linked with hyperactivity and impulsivity, both of which undermine learning ability due to its negative effect on task motivation and task persistence levels [56]. This question should be afforded focus in future studies.

The second result of this work was informing. LEV, administered throughout the entire rearing period, resulted in fewer animals presenting with LNB behavior, as evaluated against the incidence of LNB in the CTRL-exposed mice. In fact, only two animals in the LEV group, compared to 8 in the CTRL group, satisfied the criteria for LNB expression. Broadly, we regard nesting expression as a highly goal-directed behavior [39, 57, 58] that might be founded on any number of neurocognitive constructs, e.g., deficits in outcome feedback processing [36]. Here, we employed LEV to establish whether its putative beneficial effects on cognitive processes discussed in the introduction could be explored further in the present model system. It was proposed that if compulsive-like behavior is associated with neurobiological perturbations in the cortico-striatal areas [59] which associate with impaired working memory [40, 41], LEV may be a valuable drug to investigate. We have shown before that LEV engenders a level of control over the expression of HS behavior [40], i.e., decreasing the time spent engaging in stereotypy (however, see later in this work). Here, we extend these findings to the expression of LNB. Importantly, this result could not have been borne from negative effects of LEV on executive ability or motor expression since the average expression of nesting behavior was not affected (Fig. 2B). That LEV broadly influences synaptic vesicle exocytosis, without modulating the effects of a specific neurotransmitters, e.g., serotonin or dopamine, only, provides a useful point of departure for future work, especially considering the third major result of this investigation.

In contrast to effects on LNB, LEV bolstered CR (Fig. 3B), without affecting the VA scores of mice. This finding is important because it illustrates for the first time in this model system that VA and CR, as shown by a distinct subpopulation of deer mice, diverge in terms of its sensitivity for and response to pharmacological intervention, thereby potentially being founded on unique neurobiological mechanisms. Further, support for a separation of these phenotypes on the level of cognition is also provided here, since CR, but not VA intensity, correlates negatively with nesting expression (Supplementary Table IV). Interestingly, this result is likely dependent on the period of drug exposure and the age at which LEV-exposure was initiated. In our earlier work, where LEV was administered to adult mice for four weeks [40], we showed that LEV engenders a level of control over the expression of HS behavior, although at the time, no distinction was made between VA and CR. While our present findings are in line with the previous report in as far as indicating that animals exposed to LEV continues to engage in the expression of HS bouts (Fig. 3), although spending less time doing so, we show here that early intervention with LEV which is continued through the entire rearing period, bolsters the expression of CR. Interestingly, in prior clinical work, which explored the use of LEV for pediatric migraine, the drug caused hyperactivity in some patients [60]. Hyperactivity, like compulsivity and impulsivity, is associated with perturbations in cortico-striatal processes, which in turn are also responsible for motor control [61]. It is possible that CR, but not VA or LNB, shares a similar biological construct to that which may cause LEV to elicit hyperactivity.

Last, our results pertaining to the T-maze alternation behavior of deer mice did not highlight distinct differences between the alternation scores of animals in the normal, LNB and HS (irrespective of VA or CR) groupings (Supplementary Table IV). We did however show that the relationship between alternation and the time spent engaging in stereotypical behavior, was positively affected in LEV-exposed animals. In other words, the less animals engaged in stereotypical expression the more their alternation scores increased. This result seems related to the effect of LEV on the expression of VA. Although narrowly missing statistical significance, VA scores of LEV-exposed mice also trended to correlate negatively with alternation scores (Supplementary Table IV). Whereas these correlations were smaller and non-significant in CTRL-exposed animals, LEV-exposure revealed an interesting notion, i.e., that while LEV failed to elicit broad improvements on alternation, improved cognitive performance, i.e., enhanced working memory, may be seen when stereotypical engagement is positively affected. Although these associations were only modest at best, it is important to emphasize that they were not shown for LNB-expressing mice. This supports the view that stereotypy and nesting expression diverge on the level of neurocognitive architecture, but also shows that improvements in the expression of LNB scores do not contribute to working memory recall as assessed in the T-maze in the manner it was applied here. In hindsight, that LEV failed to improve working memory at large, highlights an important avenue for future investigation, especially since the exact neurocognitive mechanism of action of LEV is not yet clarified. It stands to reason that its effects on cognitive performance, rather than being robust and generalized, may be dependent on the underlying psychobiological architecture [32, 33] and how this might interact with and modify the proposed neurological effects of the drug itself.

In conclusion, we show that specific persistent behavioral phenotypes, e.g., LNB, VA and CR, are divergent in terms of their neurocognitive underpinnings and that chronic LEV administered throughout the entire rearing period may be of benefit to some phenotypes, e.g., LNB, but not others (CR). We also show that an increased level of control over the expression of stereotypy may facilitate improved working memory performance. These results are informative and pave the way for continued investigation in the deer mouse model system. Specifically, studies into the neurocognitive basis of different persistent behavioral phenotypes expressed by deer mice might be useful for our understanding of how heterogenous, but equally repetitive, behaviors spontaneously develop in specific animals of a single species only.

## Supplementary Information

Below is the link to the electronic supplementary material.

Supplementary file1 (DOCX 38 KB)

Supplementary file2 (DOCX 39 KB)

Supplementary file3 (DOCX 28 KB)

## Data Availability

The datasets generated during and/or analyzed during the current study are available from the corresponding author on reasonable request.
