# Supplementary material for: Associations between nesting, stereotypy, and working memory in deer mice: response to levetiracetam
Source: Pharmacol Rep. 2023 Apr 13;75(3):647–56. doi: 10.1007/s43440-023-00484-2 (PMC10227124; doi:10.1007/s43440-023-00484-2)
Supplement: Supplementary file 1 — Supplementary file1 (DOCX 38 KB) [file 43440_2023_484_MOESM1_ESM.docx]

Supplementary tables

**Table i - Overview of animals in the CTRL- and LEV-exposed groups that completed the respective assessments**

| Behavioral Assessment | CTRL *n* | LEV *n* |
| --- | --- | --- |
| Barnes maze | 37 | 39 |
| Nesting | 37 | 39 |
| Stereotypy* | 31 | 38 |
| T-maze | 33 | 26 |
| CTRL: control; LEV: levetiracetam; *Sample numbers are lower due to an electrical malfunction of the screening apparatus on the night of testing. Animals could not be reassessed for reasons pertaining to uniformity of methodology. | | |

**Table ii - Behavioral output generated by treatment-naïve mice selected for future CTRL-exposure across all behavioral assessments**

| Mouse | BM % success training | BM  Slope | T-maze % alternation | Nest-building | | VA | | HR | |
| --- | --- | --- | --- | --- | --- | --- | --- | --- | --- |
|  |  |  |  | **Total** | **% CV** | **Ave high** | **% Time** | **Ave high** | **% Time** |
| 1 | 37.5 | 0 | 20.0 | 34.4 | 31.0 | 795.3 | 0 | 50.0 | 0 |
| 2 | 100 | **-2.3** | 43.3 | 28.8 | 58.0 | 1243 | 0 | 19.7 | 0 |
| 3 | 62.5 | 0.9 | 37.5 | 27.9 | 55.7 | 989.7 | 0 | 16.3 | 0 |
| 4 | 62.5 | 2.1 | 42.9 | 29.4 | 30.9 | 1497.7 | 0 | 6.3 | 0 |
| 5 | 50 | **-8.1** | 30.6 | 17.2 | 55.5 | 2430.3 | 16.7 | 241.7 | 16.7 |
| 6 | 62.5 | **-1** | 62.5 | 35.0 | 46.2 | 784.3 | 0 | 5.7 | 0 |
| 7 | 87.5 | -0.4 | 42.9 | 1.8 | 66.6 | 673 | 0 | 20.7 | 0 |
| 8 | 87.5 | 0.7 | 53.8 | 12.8 | 48.7 | 1281.3 | 0 | 34.0 | 0 |
| 9 | 50 | 0.4 | 30.4 | 10.7 | 40.8 | 2152.7 | 8.3 | 37.7 | 0 |
| 10 | 50 | 0 | 18.2 | 16.3 | 43.4 | 1585 | 0 | 8.3 | 0 |
| 11 | 50 | 0 | 44.0 | 11.0 | 47.7 | 2127.3 | 8.3 | 126.0 | 0 |
| 12 | 75 | -0.5 | 39.3 | 21.6 | 32.9 | 1519.3 | 0 | 18.7 | 0 |
| 13 | 100 | **-3.8** | 20.0 | 9.8 | 61.2 | 793 | 0 | 38.0 | 0 |
| 14 | 75 | 0.3 | 36.0 | 6.7 | 57.8 | 544.3 | 0 | 18.3 | 0 |
| 15 | 50 | 0.9 | - | 19.0 | 41.3 | 2228 | 8.3 | 8.7 | 0 |
| 16 | 62.5 | 0.3 | 38.1 | 29.8 | 50.9 | 936.3 | 0 | 6.3 | 0 |
| 17 | 100 | -0.8 | 5.9 | 38.5 | 31.1 | 1550.3 | 0 | 7.3 | 0 |
| 18 | 50 | 0.4 | - | 20.4 | 28.7 | 5066.3 | 45.8 | 58.3 | 0 |
| 19 | 50 | 1 | 33.3 | 30.3 | 28.7 | 621 | 0 | 9.0 | 0 |
| 20 | 50 | -0.1 | 41.2 | 14.5 | 30.1 | - | - | - | - |
| 21 | 87.5 | **-2.2** | 35.7 | 39.4 | 27.1 | - | - | - | - |
| 22 | 50 | 0.6 | 33.3 | 13.6 | 45.2 | - | - | - | - |
| 23 | 50 | 0.2 | 58.3 | 44.4 | 12.3 | - | - | - | - |
| 24 | 50 | 0.6 | 65.6 | 30.3 | 61.6 | - | - | - | - |
| 25 | 50 | 1 | 33.3 | 33.6 | 27.1 | - | - | - | - |
| 26 | 50 | 0.8 | 43.6 | 36.7 | 25.6 | 432.3 | 0 | 32.7 | 0 |
| 27 | 50 | 1 | 55.0 | 23.0 | 42.8 | 2449.3 | 8.3 | 6.3 | 0 |
| 28 | 50 | 0.3 | - | 19.4 | 44.0 | 4483 | 33.3 | 5.7 | 0 |
| 29 | 75 | 0 | 37.5 | 34.6 | 35.5 | 1370 | 0 | 12.0 | 0 |
| 30 | 50 | -0.5 | 60.0 | 12.7 | 47.8 | 684.3 | 0 | 20.7 | 0 |
| 31 | 50 | 0.1 | 25.0 | 49.2 | 22.3 | 960.3 | 0 | 12.3 | 0 |
| 32 | 37.5 | 0.4 | 28.6 | 10.4 | 53.0 | 9041 | 58.3 | 8.7 | 0 |
| 33 | 50 | **-1.8** | 23.8 | 4.6 | 73.1 | 3586.3 | 25 | 68.0 | 0 |
| 34 | 50 | -0.9 | 21.6 | 20.0 | 52.3 | 2467.3 | 12.5 | 89.0 | 0 |
| 35 | 0 | 1.2 | - | 15.1 | 49.2 | 2216.3 | 12.5 | 44.3 | 0 |
| 36 | 50 | 0.2 | 68.8 | 22.7 | 63.0 | 1178 | 0 | 13.3 | 0 |
| 37 | 37.5 | 0.1 | 9.8 | 18.5 | 58.7 | 1451.3 | 0 | 9.0 | 0 |
| BM: Barnes maze; CV: coefficient of variance; VA: vertical jumping activity; CR: patterned cage running | | | | | | | | | |

**Table iii - Behavioral output generated by treatment-naïve mice selected for future LEV-exposure across all behavioral assessments**

| Mouse | BM % success training | BM  Slope | T-maze % alternation | Nest-building | | VA | | CR | |
| --- | --- | --- | --- | --- | --- | --- | --- | --- | --- |
|  |  |  |  | **Total** | **% CV** | **Ave high** | **% Time** | **Ave high** | **% Time** |
| 38 | 75 | -0.9 | 80.0 | 21.4 | 48.1 | - | - | - | - |
| 39 | 50 | 0 | 34.5 | 39.0 | 36.6 | 1084.3 | 0 | 43.7 | 0 |
| 40 | 50 | 0 | 11.8 | 28.1 | 32.5 | 1392.7 | 0 | 19.7 | 0 |
| 41 | 12.5 | -0.3 | - | 22.8 | 31.5 | 209.7 | 0 | 33.3 | 0 |
| 42 | 50 | 0.5 | - | 20.8 | 48.1 | 240.3 | 0 | 364.7 | 41.7 |
| 43 | 50 | 0.4 | 45.2 | 46.8 | 24.2 | 177.3 | 0 | 113.3 | 0 |
| 44 | 100 | -0.1 | 51.9 | 21.5 | 27.9 | 1250 | 0 | 16.3 | 0 |
| 45 | 50 | -0.8 | 17.6 | 28.7 | 36.5 | 1677 | 4.2 | 38.0 | 0 |
| 46 | 50 | 0 | - | 25.6 | 41.3 | 921 | 0 | 14.3 | 0 |
| 47 | 25 | 0.3 | - | 0.5 | 327.0 | 2718 | 29.2 | 258.0 | 8.3 |
| 48 | 50 | 0.1 | 44.0 | 14.8 | 30.3 | 859.7 | 0 | 4.0 | 0 |
| 49 | 37.5 | 0.6 | 25.0 | 10.8 | 28.7 | 1075.3 | 0 | 16.7 | 0 |
| 50 | 50 | 0.4 | 47.2 | 22.9 | 39.2 | 649.7 | 0 | 48.3 | 0 |
| 51 | 37.5 | 0 | - | 33.6 | 53.4 | 339.7 | 0 | 10.7 | 0 |
| 52 | 50 | -0.6 | - | 16.7 | 40.2 | 979 | 0 | 27.0 | 0 |
| 53 | 12.5 | -0.3 | - | 15.3 | 26.0 | 1349.3 | 0 | 162.7 | 4.2 |
| 54 | 87.5 | 0.7 | - | 20.9 | 32.8 | 1863.7 | 4.2 | 36.0 | 0 |
| 55 | 87.5 | -0.7 | 20.0 | 19.0 | 22.4 | 633.3 | 0 | 30.7 | 0 |
| 56 | 50 | -0.5 | 21.2 | 20.6 | 51.3 | 996 | 0 | 34.7 | 0 |
| 57 | 87.5 | -0.2 | 46.7 | 27.3 | 15.8 | 1534 | 0 | 17.7 | 0 |
| 58 | 50 | 0.3 | - | 14.6 | 29.7 | 2080.7 | 4.2 | 34.7 | 0 |
| 59 | 62.5 | 0.4 | 46.2 | 16.0 | 36.6 | 763 | 0 | 18.7 | 0 |
| 60 | 62.5 | -0.8 | 33.3 | 15.4 | 47.8 | 2025.7 | 8.3 | 20.3 | 0 |
| 61 | 37.5 | -0.1 | 11.8 | 22.2 | 45.0 | 2390.3 | 12.5 | 6.3 | 0 |
| 62 | 50 | 0.1 | 31.4 | 18.3 | 37.6 | 795.7 | 0 | 566.7 | 80.3 |
| 63 | 50 | 0 | 19.4 | 17.3 | 44.9 | 1196.3 | 0 | 12.7 | 0 |
| 64 | 62.5 | -0.1 |  | 24.3 | 27.3 | 1332 | 0 | 11.7 | 0 |
| 65 | 62.5 | -0.4 | 24.0 | 11.9 | 47.2 | 182.3 | 0 | 72.3 | 0 |
| 66 | 50 | -0.5 |  | 18.2 | 39.9 | 2265.7 | 8.3 | 31.3 | 0 |
| 67 | 62.5 | 0.4 | 16.7 | 18.1 | 56.0 | 2808.7 | 16.7 | 66.0 | 0 |
| 68 | 50 | -0.5 | 43.5 | 7.0 | 68.1 | 1101 | 0 | 161.0 | 0 |
| 69 | 50 | -0.1 | 28.6 | 18.6 | 44.4 | 3962 | 29.2 | 66.0 | 0 |
| 70 | 50 | -0.4 | 60.0 | 5.6 | 75.7 | 1234.7 | 0 | 125.0 | 0 |
| 71 | 87.5 | 0.5 |  | 1.6 | 128.2 | 402 | 0 | 74.7 | 0 |
| 72 | 50 | 0.6 | 42.4 | 11.6 | 58.4 | 1795 | 4.2 | 47.7 | 0 |
| 73 | 62.5 | -0.9 | 29.0 | 31.9 | 39.6 | 937.3 | 0 | 14.0 | 0 |
| 74 | 50 | 0.1 |  | 4.2 | 62.4 | 3132 | 16.7 | 378.0 | 50 |
| 75 | 50 | 0.8 | 56.3 | 4.6 | 71.4 | 715.7 | 0 | 24.7 | 0 |
| 76 | 50 | 1.1 | 55.6 | 32.9 | 20.8 | 652 | 0 | 88.3 | 0 |
| BM: Barnes maze; CV: coefficient of variance; VA: vertical jumping activity; CR: patterned cage running | | | | | | | | | |

**Table iv - Spearman's correlations between the indicated measures collected in adulthood**

| Correlation | Group | Spearman’s *r* [CI] | *p* | Pairs *n* |
| --- | --- | --- | --- | --- |
| Total nesting score (g) vs. VA intensity | CTRL | -0.246 [-0.597; 0.295] | 0.182 | 31 |
|  | LEV | -0.2196  [-0.511; 0.117] | 0.185 | 38 |
| Total nesting score (g) vs. CR intensity | CTRL | -0.4236  [-0.68; -0.07] | **0.018*** | 31 |
|  | LEV | -0.3063  [-0.577; 0.025] | 0.062 | 38 |
| Total nesting score (g) vs. % time spent HS | CTRL | -0.3916  [-0.661; -0.032] | **0.029*** | 31 |
|  | LEV | -0.2411  [-0.528; 0.095] | 0.145 | 38 |
| Total nesting score (g) vs. % T-maze alternation | CTRL | 0.086  [-0.269; 0.421] | 0.627 | 34 |
|  | LEV | 0.017  [-0.383; 0.412] | 0.935 | 26 |
| VA intensity vs. % T-maze alternation | CTRL | -0.282 [-0.606; 0.121] | 0.154 | 27 |
|  | LEV | -0.349  [-0.661; 0.066] | 0.087 | 25 |
| CR intensity vs.  % T-maze alternation | CTRL | -0.089  [-0.463; 0.312] | 0.658 | 27 |
|  | LEV | 0.210  [-0.213; 0.568] | 0.313 | 25 |
| % Time spent HS vs.  % T-maze alternation | CTRL | -0.144  [-0.506; 0.261] | 0.474 | 27 |
|  | LEV | -0.396  [-0.691; 0.011] | **0.049*** | 25 |
| CTRL: control; LEV: levetiracetam; VA: vertical jumping activity; CR: patterned cage running; HS: high stereotypical behavior | | | | |
