# Supplementary material for: Associations between nesting, stereotypy, and working memory in deer mice: response to levetiracetam
Source: Pharmacol Rep. 2023 Apr 13;75(3):647–56. doi: 10.1007/s43440-023-00484-2 (PMC10227124; doi:10.1007/s43440-023-00484-2)
Supplement: Supplementary file 2 — Supplementary file2 (DOCX 39 KB) [file 43440_2023_484_MOESM2_ESM.docx]

Supplementary drug intake data

Here, a graphical representation of the drug intake data of twelve animals per exposure group (water and levetiracetam; LEV) are shown. Drug intake remained consistent with age throughout the rearing period, while the fluid intake of mice of the different exposure groups, did not differ. Please refer to the MS for more detail.

The average daily cage fluid measurement of 24 mice in 12 cages (two mice per cage, aged 12 weeks) that received either normal water or levetiracetam solution. Two-way RM ANOVA. No significant interactions or main effects shown.
