# Supplementary material for: Associations between nesting, stereotypy, and working memory in deer mice: response to levetiracetam
Source: Pharmacol Rep. 2023 Apr 13;75(3):647–56. doi: 10.1007/s43440-023-00484-2 (PMC10227124; doi:10.1007/s43440-023-00484-2)
Supplement: Supplementary file 3 — Supplementary file3 (DOCX 28 KB) [file 43440_2023_484_MOESM3_ESM.docx]

Supplementary methodological detail

In this supplementary document, detailed descriptions of the methods followed in this work, are provided. For any other queries, these can be directed to the corresponding author.

***Barnes maze assessment***

All BM experiments were conducted in the dark cycle, between 19:00 and 01:00. A maximum of 15 mice were assessed on any given night. To allow animals to fully wake, the first cages were moved to the experimental room at 18:00. After this, animals were moved to the testing room in a sequential manner, on any given night of testing.

A small, walled-off Barnes maze, adapted from O'Leary and Brown (47), was used. The maze was constructed from white Plexiglas^®^ and consisted of an octagon-shaped floor with a diameter of 70 cm, walled off on each side (50 cm high) to prevent the influence of external cues on learning, as far as possible. 16 circular holes (4 cm in diameter) were equally spaced around the perimeter of the maze floor (holes were spaced 3 cm from the walls). The maze was raised to a height of 30 cm above floor level to allow for the placement of a movable escape box (15 cm (l) x 15 cm (w) x 20 cm (h)). The escape box was constructed from black Plexiglas^®^ and connected to a pipe (Ø = 7 cm) that could be attached to any of the holes to provide a means of escape. Before each trial, the escape box was cleaned with 90 % ethanol, rinsed with normal tap water, and allowed to dry, before being prepared with corn cob bedding and food taken from the respective home cages of the tested mice. When the escape box was attached to the BM, all other holes were closed off with black lids placed at a depth reaching 2 cm below the floor level of the maze. A separate floorless starting compartment (10 cm x 10 cm x 15 cm) was constructed from black Plexiglas^®^. This was placed in the center of the BM at the onset of testing. Once experimentation commenced, the starting compartment was removed from the arena, and the mouse left to freely explore. Except if stated otherwise, all phases of the Barnes maze experiment were conducted under bright white light (10 000 lux), while white noise was played in the background at a sound level of 80 dB to bolster escape-related behavior (48).

The BM assessment was conducted as previously described (47), with minor modification to allow for the assessment of juvenile deer mice. Briefly, mice were habituated, trained, and tested over three separate stages: **(A)** habituation stage, **(B)** acquisition stage, and **(C)** probe stage. All trials were video recorded to allow for post-test scoring and automated tracking of ambulatory behavior (Ethovision^®^ XT 16; Noldus Information Technology^®^, Wageningen, The Netherlands).

For the habituation stage **(A)** (experimental day 1, PND 28), mice were introduced to the BM for two 5-min trials spaced 25 min apart. The first habituation was conducted under dim red light without playing white background noise to allow for habituation to the maze under less stressful conditions. Mice were transferred to the BM and placed underneath a 2 L glass beaker located over the hole where the escape burrow was located. The escape box was attached to the hole underneath the glass beaker. Mice were thus able to view the entire spatial environment, but only had access to the hole connected to the escape burrow and box. Next, mice were once again transferred to the BM; however, at this time, mice were placed in the middle of the BM underneath the starting compartment located in the center of the maze and left there for one minute. This session was also conducted under dim red light and in the absence of background noise. After the starting compartment was raised, mice were allowed five minutes to explore the entire maze and find the escape hole with all the other holes still being closed off.

The acquisition stage **(B)** comprised four training days (PND 29-32; two trials per day, spaced 25 min apart). At the beginning of each trial, mice were introduced to starting compartment located at the center of the maze for 1 min. On the first day, the maze was brightly lit only with the trials conducted in the absence of white noise. Over the second to fourth days of stage **(B)**, background noise was added to the testing procedure. Again, mice had 5 min to explore the maze during each trial and to find the escape hole, which was still located in the same position as in the habituation phase. Successful target acquisition in each trial was accepted if mice spent at least 5 sec in the proximity of target hole (within 5 cm of its border) or when mice entered the escape box. Once reaching the escape box, mice were allowed to remain there for 30 sec before being returned to their home cages. Mice that spent time at the escape hole, but that did not enter the burrow, were left in the BM for the full 5-min duration. Irrespective of the number of times said animals returned to the proximity area around the escape hole, learning was counted only once per trial. After completing each trial, the maze and the escape box were cleaned as explained for stage **(A)**. Mice were accepted to have learned the location of the escape hole if they made four or more successful attempts at reaching the hole or entered the burrow over the eight stage **(B)** trials, collectively.

In the probe stage **(C)**, animals were assessed for habit-like behavior, i.e. returning to the proximity area around, or attempting to enter the hole to which the escape box was previously attached, but was now closed off. The probe trial phase (PND 33-34; 2 trials per day) was conducted over 2 days of testing. The first probe trail began 24 h after the last stage **(B)** trial. Here, we quantified the number of approaches made towards the target area during each trial. More entries towards the target area in the first trial compared to the last, was regarded as behavioral engagement regulated by goal-directed processing, while a similar number of approaches towards the target area through the final stage **(C)** trial, was regarded as habit-like. These observations were informed by negative and neutral-to-positive trial vs. target approach-slopes generated by each mouse, respectively. Mice generating slopes < -1 were defined as goal-directed because they modified their response strategies when the escape box was occluded, while scores > -1 were considered habit-like because mice persisted in the same routine.

***Nesting assessment***

All 76 mice were assessed for nest building expression over one week once animals reached the age of 84 days (34). For this assessment, each mouse was allocated to its own new home cage, identical to the home cage, for seven days. On each of the seven days of nesting assessment, between 15:00 and 16:00, an excess of sterile and unscented cotton wool was weighed and introduced into the roof of each cage. On every subsequent day, also between 15:00 and 16:00, built nests were removed, discarded and the remaining cotton wool in the roof of the cage weighed and recorded. Where needed, additional cotton wool was supplied. Thus, mice had access to nesting material for 24 h of each day.

After completion of the 7-day nesting assessment, the daily quantity of cotton wool that was utilized by each animal (in grams) was summed and a 7-day total nesting score calculated for each subject (34). Identification of normal nesting (NNB) and LNB (where applicable) was based on the extreme ends of the distribution of all total nesting scores generated by mice in the CTRL- and LEV-exposed groups (34). Further, a second criterion, that is persistence, was applied to classify LNB. Thus, only animals that generated total nesting scores that clustered within the upper 75^th^ percentile of the distribution, and the lowest quartile of distribution with respect to the variance in the daily nesting scores (as reflected by the percentage coefficient of variance; % CV), are classified as LNB-expressing animals (**Fig. 2A**). Conversely, NNB animals were identified as those mice of which the nesting scores clustered between the 25^th^ and 50^th^ percentile of the total nesting score distribution (34). During periods of nest building analysis, food and water were available as normal, although animals were not provided with any additional form of nesting material.

***Stereotypy assessment***

On the first day following the conclusion of the nest building assessment, all mice were assessed for stereotypical behavior according to a previously published protocol (40), slightly modified for the purposes of the present investigation. Briefly, each mouse underwent a single 12-h stereotypy assessment. At 17:00, one hour prior to the onset of screening, animals were moved in their housing cages to the behavioral screening room located on the same floor of the vivarium. Each animal was then immediately introduced to its own behavioral testing cage [21 cm (l) x 21 cm (w) x 35 cm (h); Accuscan^®^ Inc., Columbus, Ohio, USA], that was constructed from clear Plexiglas^®^ and equipped with a grid of infrared beams that crossed the cage at both 2 cm and 10 cm above floor level. Ground corncob was provided in quantities enough to cover the floor of the test cages, but also ensuring that the scoring of ambulatory activity was not interrupted. Food and water (or drug solutions) were again provided *ad lib* throughout the 12-h assessment session. Cages were cleaned with F10^®^ veterinary cleaner (Health and Hygiene Products Pty. Ltd., Roodepoort, South Africa) after each night of assessment and prepared for the next assessment session.

Infrared beam tracking allows for the quantification of both VA and pattern running (expressed as the sum of all clockwise and anticlockwise CR. Since mice that express jumping activity interrupt more than one beam in the higher infrared grid when making a single jump (43), the number of vertical beam interruptions is applied as broad measure of VA. Where applicable, mice were classified as either non-stereotypical (NS) or HS based on two measures, i.e. behavioral intensity and the time spent executing HS behavior. To do this, the 12-h screening session was divided into 24 30-min bouts. HS bouts are defined for each behavioral phenotype as per the cut-off values in **Table 1**. Stereotypical intensity was calculated as the average of the three highest 30-min VBI or CR values generated, whereas time spent engaging in stereotypy was calculated as the number of 30-min HS bouts expressed during the 12-h screening session by each mouse, expressed as a percentage. Importantly, HS animals were selected based on the expression of either vertical or horizontal activity, or both.

**Table 1 - Cut-off criteria for NS and HS behavior across both behavioral phenotypes**

| Cohort | VBI / 30 min | CR / 30 min |
| --- | --- | --- |
| NS | < 500 | < 150 |
| HS | > 2000 | > 200 |
| NS: non-stereotypical; HS: high stereotypical; VBI: vertical beam interruptions (indicative of vertical jumping activity); CR: cage revolutions (sum of clockwise and anti-clockwise revolutions) | | |

***T-maze***

Assessment of arm alternation behavior in a T-maze is often used to investigate processes related to working memory (49). Here, we applied a modified version of the test used by (50); however, in this work, the walls of the maze was raised to accommodate for the screening of jumping deer mice. Animals were also trained to enter one arm of the maze, prior to alternation testing, to test for habit-like behavior. Briefly, the maze, shaped in the form the letter ‘T’, was constructed from white, opaque Plexiglas^®^ (stem and arm dimensions 30 cm x 6.5 cm; wall height: 30 cm). When needed (see below), each of the respective arms could be closed off by a manually operated door (51). A removable divider placed 10 cm from the base of the stem, was used to confine animals to a ‘starting area’. The ambulatory activity of mice was analyzed with Ethovision^®^ XT 16 software. Following each T-maze assessment session, the maze was cleaned with 90 % ethanol, rinsed with normal tap water and dried to prevent the possible influence of previously associated odors on the alternation behavior of subsequently assessed mice (50).

The T-maze assessment was conducted over three stages**:** **(A)** naturalistic arm preference testing, **(B)** cued forced-arm entry training, and **(C)** probe test. Stages were separated by 24 h, with stage **(A)** commencing 24 h after the onset of stereotypy assessment. A separate habituation phase was not included as in the BM assessments since animals were allowed to explore the entire arena during the naturalistic arm preference phase. All T-maze experiments were conducted between 19:00 and 01:00 under dim red light and videotaped. Mice were moved from the housing environment to the testing room on the same floor of the vivarium at least one hour prior to the onset of testing.

Naturalistic arm preference testing was performed on PND 93 (52). Mice were allowed a single 15-min session to explore the maze, or to make a maximum of ten arm entries. The most preferred arm was determined based on the majority arm choice. Animals that failed to make ten arm entries within the 15-min window were not included for assessment in stages **(B)** and **(C)**. For animals that made an equal number of left- or right choices, an arm choice was randomly chosen by the observer for application in stage **(B)**. Stage **(A)** began when an animal was introduced to the enclosed starting area. After 30 sec, the starting area door was raised and animals were allowed to make their first entry into any one of the transverse arms of the maze (52). Following the choice execution, the opposite arm was closed off and the animals allowed to freely return to the starting area. Here, mice were confined for 30 sec (52), before being allowed to enter the maze again.

For the cued forced-arm entry stage **(B)**, which was conducted when mice were aged PND 94, animals were forced to enter the least preferred arm (i.e. ‘forced arm’) as determined in stage **(A)**. In this instance, the forced arm was cued on all three sides with a solid black rectangular mark to promote arm recognition during stage **(C)**. Animals were allowed to make as many arm entries as they could within a single 30-min session. However, animals that completed less than ten arm entries were not included for assessment in stage **(C)**.

During the probe test stage **(C)** (PND 95), mice were able to access the entire arena again. The arm that was cued in stage **(B)** remained thus, with the opposite arm being non-cued. Again, mice were allowed to explore the maze and make as many arm choices as they could for 30 min during a single session, with an intertrial session again set at 30 sec. Arm choices were noted and the alternation score, expressed as a percentage of the total arm entries, calculated. Higher, as opposed to lower post-training alternation scores are indicative of better working and spatial memory recall.
